# Supplementary material for: Sustained-input switches for transcription factors and microRNAs are central building blocks of eukaryotic gene circuits
Source: Genome Biol. 2013 Aug 23;14(8):R85. doi: 10.1186/gb-2013-14-8-r85 (PMC4054853; doi:10.1186/gb-2013-14-8-r85)
Supplement: Additional file 5 — HTML Browsable Motif Output. Zipped folder containing all WaRSwap and FANMOD motif output, viewable in a web browser. [file gb-2013-14-8-r85-S5.ZIP › HTML_browsable_motif_output/FANMOD_ath_tair9/sigs_fanmodm-2000.pvals.heatmaps.html/motif_id_46_011101001_tftype_ath_upstream_-3000_0.html]

```
BG_MODEL = FANMOD
MOTIF_ID = 46_011101001
TF_TYPE = ath
UPSTREAM = -3000_0


PVals
FN_0.2	FN_0.4	FN_0.6	FN_0.8
dg_60.genes	0.187	0.04	0.008	0.809
dg_70.genes	0.175	0.045	0.004	0.828
dg_80.genes	0.174	0.033	0.011	0.831

ZScores
FN_0.2	FN_0.4	FN_0.6	FN_0.8
dg_60.genes	0.909	1.76	2.372	-1.297
dg_70.genes	0.918	1.718	2.458	-1.359
dg_80.genes	0.933	1.809	2.428	-1.341

StDevs
FN_0.2	FN_0.4	FN_0.6	FN_0.8
dg_60.genes	165.779	206.89	71.361	13.083
dg_70.genes	163.681	205.332	69.142	13.251
dg_80.genes	169.509	207.775	70.102	13.413
```
